# WHAT DO OLDER ADULTS THINK ABOUT ELECTRONIC PAIN APPS? USING A HEALTH LITERACY FRAMEWORK TO MEASURE QUALITY

**DOI:** 10.1093/geroni/igad104.2825

**Published:** 2023-12-21

**Authors:** Mary Milidonis, Hayley Rupp, Patrick McGinty, Karen Kopiera-Frye

**Affiliations:** Cleveland State University, Cleveland, Ohio, United States; Cleveland State University, Cleveland, Ohio, United States; Cleveland State University, Cleveland, Ohio, United States; New Mexico State University, Las Cruces, New Mexico, United States

## Abstract

Mobile pain applications (apps) may improve health communication. Mobile health apps do not always consider the needs of older adults. This project examines perceptions of pain apps on smartphones among older adults. The sample consisted of 23 older adults (M age = 64 years), with majority female (83%), predominantly Caucasian (91%), and majority some college education (65%). Respondents answered questions about 5 free pain apps using the Mobile Application Rating Scale (MARS). The MARS assesses descriptive and technical aspects of an eHealth apps using five categories including four quality scales: engagement, functionality, aesthetics, and information and one subjective quality scale. Participants currently had pain with range of 3 months to 12 years. The Single Item Health Literacy Screener identified 17 people have never asked for help reading health information; all participants scored 100% on the Rapid Estimate of Adult Literacy in Medicine Short Form. Participants self-reported an average of 2.6 comorbidities on the Functional Comorbidity Scale. The participants overall MARS rating ranged from 1-5; 11 participants rated pain apps very high (4-5). Important app characteristics identified by the older adults as critical for useable app included: Clear instructions, good navigation, and easy functionality. The elders reported that difficulty with loading the app and slow times would deter their use of the app. In terms of frequency of use, participants felt using the app was important weekly only if they experienced pain. These are important considerations in designing age-friendly technology for use with the frequent condition of pain among older adults.

